# 4,4′-(2,6-Dihydroxy­naphthalene-1,5-diyldimethyl­ene)dipyridinium bis­(per­chlorate)

**DOI:** 10.1107/S1600536808015092

**Published:** 2008-05-30

**Authors:** Wei-Feng Zhu, Zheng Xing

**Affiliations:** a School of Materials Science and Engineering, Jiangsu University of Science and Technology, Zhenjiang, Jiangsu 212003, People’s Republic of China

## Abstract

The title compound, C_22_H_20_N_2_O_2_^2+^·2ClO_4_^−^, was synthesized by the reaction of naphthalene-2,6-diol with pyridine-4-carbaldehyde, 4-picolylamine and perchloric acid. There is a centre of symmetry at the mid-point of the central C—C bond of the cation. The two pyridine rings are parallel to each other, and the dihedral angle between the naphthalene ring system and the pyridine ring is 80.68 (11)°. All the bond lengths and angles are normal. Classical inter­molecular O—H⋯O and N—H⋯O hydrogen bonds connect cations and anions, forming a one-dimensional chain structure.

## Related literature

For related literature, see: Fu & Zhao (2007 ▶); Aoki *et al.* (2004 ▶); Jacobsson & Ellervik (2002 ▶); Sasada *et al.* (2003 ▶); Szatmári *et al.* (2003 ▶); Szatmári *et al.* (2004 ▶); Cardellicchio *et al.* (1999 ▶). For a comparison of bond lengths and angles, see: Oloo *et al.* (2002 ▶).
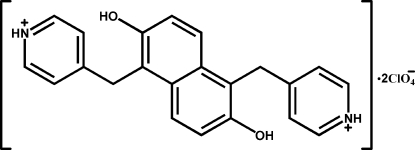


## Experimental

### 

#### Crystal data

C_22_H_20_N_2_O_2_^2+^·2ClO_4_^−^
*M* *_r_* = 543.30
Monoclinic, 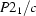

*a* = 4.9587 (4) Å
*b* = 13.0399 (11) Å
*c* = 17.8291 (16) Å
β = 96.767 (2)°
*V* = 1144.82 (17) Å^3^
*Z* = 2
Mo *K*α radiation
μ = 0.35 mm^−1^
*T* = 296 (2) K
0.30 × 0.20 × 0.05 mm

#### Data collection

Bruker SMART APEX CCD area-detector diffractometer
Absorption correction: multi-scan (*SADABS*; Bruker, 2000 ▶) *T*_min_ = 0.925, *T*_max_ = 0.988
6156 measured reflections
2011 independent reflections
1610 reflections with *I* > 2σ(*I*)
*R*_int_ = 0.023

#### Refinement

*R*[*F* ^2^ > 2σ(*F* ^2^)] = 0.059
*wR*(*F* ^2^) = 0.151
*S* = 1.06
2011 reflections
168 parameters
H-atom parameters constrained
Δρ_max_ = 0.46 e Å^−3^
Δρ_min_ = −0.27 e Å^−3^

### 

Data collection: *SMART* (Bruker, 2000 ▶); cell refinement: *SAINT* (Bruker, 2000 ▶); data reduction: *SAINT*; program(s) used to solve structure: *SHELXS97* (Sheldrick, 2008 ▶); program(s) used to refine structure: *SHELXL97* (Sheldrick, 2008 ▶); molecular graphics: *SHELXTL* (Sheldrick, 2008 ▶); software used to prepare material for publication: *SHELXTL*.

## Supplementary Material

Crystal structure: contains datablocks I, global. DOI: 10.1107/S1600536808015092/wn2262sup1.cif

Structure factors: contains datablocks I. DOI: 10.1107/S1600536808015092/wn2262Isup2.hkl

Additional supplementary materials:  crystallographic information; 3D view; checkCIF report

## Figures and Tables

**Table 1:** Hydrogen-bond geometry (Å, °)

*D*—H⋯*A*	*D*—H	H⋯*A*	*D*⋯*A*	*D*—H⋯*A*
O1—H1*C*⋯O2^i^	0.82	2.05	2.859 (4)	169
N1—H1*B*⋯O3^ii^	0.86	2.24	2.997 (4)	148
N1—H1*B*⋯O4^ii^	0.86	2.33	3.121 (5)	153

Symmetry codes: (i) 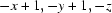; (ii) 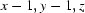.

**Fig. 1.**
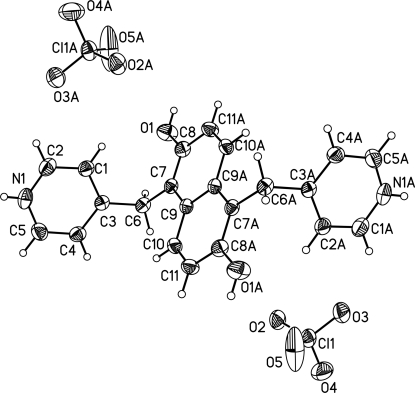
A view of the title compound with the atomic numbering scheme. Displacement ellipsoids are drawn at the 30% probability level. Symmetry code (A): -x, -y + 1, -z.

**Fig. 2.**
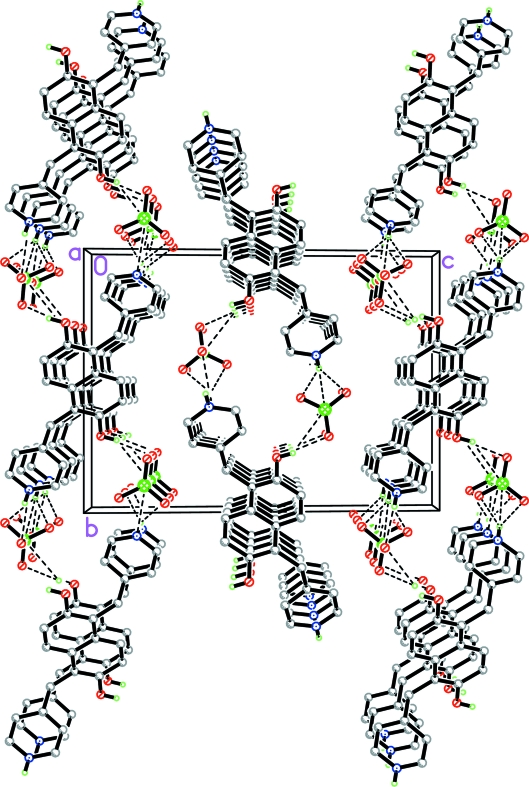
The packing of the structure, viewed down the a axis, showing molecules connected by O—H···O and N—H···O hydrogen bonds (dashed lines). H atoms not involved in hydrogen bonding have been omitted.

## supplementary crystallographic information

### Comment

Phenols and naphthols are an important class of compounds for the syntheses of dyes, pharmaceuticals and polymers. In particular, naphthalenediols are essential components of intelligent polymers such as engineering plastics and liquid crystalline polymers. 2,6-Naphthalenediol has attracted much attention for its chemical and physical properties as a liquid crystalline monomer material (Aoki *et al., *2004; Jacobsson & Ellervik, 2002; Sasada *et al.*, 2003). Electron-rich naphthols are also known to be good C-nucleophiles with the ability to undergo ready addition to C═N double bonds in modified Mannich condensations (Szatmári *et al.*, 2003; Szatmári *et al.*, 2004 and Cardellicchio *et al.*,1999). A similar 1,1'-binaphthyl derivative has been reported recently (Fu & Zhao, 2007).

The structure of the title compound is illustrated in Fig. 1. All the bond lengths and angles are normal (Oloo *et al.*, 2002). The two pyridine rings are parallel to each other, and the dihedral angle between the naphthol ring system and the pyridine ring is 80.68 (11)°. The C3—C6—C7—C9 torsion angle is 82.8 (3)°. The packing diagram (Fig. 2) shows that three classical intermolecular O—H···O and N—H···O hydrogen-bonds (Table 1) link cations and anions to form a one-dimensional chain structure.

### Experimental

Naphthalene-2,6-diol (1.60 g, 10 mmol), pyridine-4-carbaldehyde (1.07 g, 10 mmol), 4-picolylamine (1.08 g, 10 mmol) and perchloric acid (3 ml) were well mixed and heated to 120°C, cooled down and 25 ml ethanol was added after TLC showed that the reaction was complete. Well dispersed by ethanol, 1.50 g white powder was collected after filtration and finally recrystallized from ethanol, yielding the yellow title compound.

### Refinement

H atoms bonded to O and N atoms were located in a difference map and refined with distance restraints of O—H = 0.82 and N—H = 0.86 Å, and with *U*_iso_(H) = 1.5*U*_eq_(O,*N*). Other H atoms were positioned geometrically and were allowed to ride on the C atoms to which they are bonded, with C—H = 0.93–0.97 Å; *U*_iso_(H) = x*U*_eq_(C), where x= 1.5 for Csp^2^ and 1.2 for Csp^3^.

### Crystal data

**Table:**

C_22_H_20_N_2_O_2_^2+^·2ClO_4_^–^	*F*_000_ = 560
*M**_r_* = 543.30	*D*_x_ = 1.576 Mg m^−^^3^
Monoclinic, *P*2_1_/*c*	Mo *K*α radiation λ = 0.71073 Å
Hall symbol: -P 2ybc	Cell parameters from 3755 reflections
*a* = 4.9587 (4) Å	θ = 2.8–25.0º
*b* = 13.0399 (11) Å	µ = 0.35 mm^−^^1^
*c* = 17.8291 (16) Å	*T* = 296 (2) K
β = 96.767 (2)º	Tabular, yellow
*V* = 1144.82 (17) Å^3^	0.30 × 0.20 × 0.05 mm
*Z* = 2	

### Data collection

**Table:**

Bruker SMART APEX CCD area-detector diffractometer	2011 independent reflections
Radiation source: fine-focus sealed tube	1610 reflections with *I* > 2σ(*I*)
Monochromator: graphite	*R*_int_ = 0.023
*T* = 296(2) K	θ_max_ = 25.0º
φ and ω scans	θ_min_ = 2.8º
Absorption correction: multi-scan(SADABS; Bruker, 2000)	*h* = −5→5
*T*_min_ = 0.925, *T*_max_ = 0.988	*k* = −14→15
6156 measured reflections	*l* = −21→14

### Refinement

**Table:**

Refinement on *F*^2^	Secondary atom site location: difference Fourier map
Least-squares matrix: full	Hydrogen site location: inferred from neighbouring sites
*R*[*F*^2^ > 2σ(*F*^2^)] = 0.059	H-atom parameters constrained
*wR*(*F*^2^) = 0.151	*w* = 1/[σ^2^(*F*_o_^2^) + (0.0742*P*)^2^ + 0.8634*P*] where *P* = (*F*_o_^2^ + 2*F*_c_^2^)/3
*S* = 1.07	(Δ/σ)_max_ < 0.001
2011 reflections	Δρ_max_ = 0.46 e Å^−^^3^
168 parameters	Δρ_min_ = −0.27 e Å^−^^3^
Primary atom site location: structure-invariant direct methods	Extinction correction: none

### Special details

**Table:**

Geometry. All e.s.d.'s (except the e.s.d. in the dihedral angle between two l.s. planes) are estimated using the full covariance matrix. The cell e.s.d.'s are taken into account individually in the estimation of e.s.d.'s in distances, angles and torsion angles; correlations between e.s.d.'s in cell parameters are only used when they are defined by crystal symmetry. An approximate (isotropic) treatment of cell e.s.d.'s is used for estimating e.s.d.'s involving l.s. planes.
Refinement. Refinement of *F*^2^ against ALL reflections. The weighted *R*-factor *wR* and goodness of fit *S* are based on *F*^2^, conventional *R*-factors *R* are based on *F*, with *F* set to zero for negative *F*^2^. The threshold expression of *F*^2^ > σ(*F*^2^) is used only for calculating *R*-factors(gt) *etc*. and is not relevant to the choice of reflections for refinement. *R*-factors based on *F*^2^ are statistically about twice as large as those based on *F*, and *R*- factors based on ALL data will be even larger.

### Fractional atomic coordinates and isotropic or equivalent isotropic displacement parameters (Å^2^)

**Table:**

	*x*	*y*	*z*	*U*_iso_*/*U*_eq_	
C1	−0.1573 (8)	0.1073 (3)	0.0739 (2)	0.0616 (10)	
H1A	−0.2367	0.0654	0.0352	0.074*	
C2	0.0188 (7)	0.1821 (2)	0.0583 (2)	0.0529 (8)	
H2A	0.0589	0.1915	0.0092	0.063*	
C3	0.1388 (6)	0.2445 (2)	0.11614 (17)	0.0407 (7)	
C4	0.0721 (7)	0.2263 (3)	0.18810 (19)	0.0532 (8)	
H4A	0.1488	0.2662	0.2283	0.064*	
C5	−0.1058 (8)	0.1501 (3)	0.2003 (2)	0.0630 (10)	
H5A	−0.1492	0.1378	0.2488	0.076*	
C6	0.3317 (6)	0.3289 (2)	0.10067 (18)	0.0456 (8)	
H6A	0.5071	0.2991	0.0947	0.055*	
H6B	0.3558	0.3746	0.1439	0.055*	
C7	0.2348 (6)	0.3905 (2)	0.03090 (17)	0.0412 (7)	
C8	0.3277 (6)	0.3667 (2)	−0.03634 (19)	0.0489 (8)	
C9	0.0446 (6)	0.4723 (2)	0.03359 (17)	0.0390 (7)	
C10	−0.0624 (6)	0.4991 (2)	0.10063 (17)	0.0462 (8)	
H10A	−0.0082	0.4628	0.1447	0.055*	
C11	−0.2443 (7)	0.5773 (3)	0.10207 (19)	0.0531 (8)	
H11A	−0.3133	0.5936	0.1469	0.080*	
Cl1	0.28576 (17)	0.89190 (6)	0.17150 (5)	0.0565 (3)	
N1	−0.2164 (6)	0.0937 (2)	0.1433 (2)	0.0618 (8)	
H1B	−0.3305	0.0466	0.1518	0.093*	
O1	0.5047 (5)	0.2863 (2)	−0.03716 (15)	0.0732 (8)	
H1C	0.5186	0.2701	−0.0810	0.110*	
O2	0.3960 (6)	0.79156 (19)	0.18131 (15)	0.0704 (8)	
O3	0.3505 (7)	0.9360 (2)	0.10369 (15)	0.0823 (9)	
O4	0.4082 (10)	0.9556 (3)	0.22951 (18)	0.1228 (15)	
O5	0.0089 (7)	0.8878 (3)	0.1717 (4)	0.177 (3)	

### Atomic displacement parameters (Å^2^)

**Table:**

	*U*^11^	*U*^22^	*U*^33^	*U*^12^	*U*^13^	*U*^23^
C1	0.072 (2)	0.0445 (19)	0.065 (2)	0.0003 (17)	−0.007 (2)	−0.0014 (17)
C2	0.066 (2)	0.0429 (18)	0.0483 (19)	0.0033 (15)	−0.0006 (17)	0.0020 (15)
C3	0.0432 (16)	0.0336 (15)	0.0431 (17)	0.0128 (12)	−0.0042 (13)	0.0051 (13)
C4	0.063 (2)	0.0491 (19)	0.0459 (19)	0.0051 (16)	−0.0017 (16)	−0.0011 (15)
C5	0.075 (3)	0.058 (2)	0.058 (2)	0.0047 (19)	0.015 (2)	0.0109 (18)
C6	0.0447 (17)	0.0406 (16)	0.0491 (19)	0.0056 (13)	−0.0049 (14)	0.0010 (14)
C7	0.0426 (16)	0.0362 (15)	0.0432 (17)	0.0003 (13)	−0.0020 (13)	0.0031 (13)
C8	0.0473 (18)	0.0421 (17)	0.057 (2)	0.0121 (14)	0.0068 (15)	0.0009 (15)
C9	0.0389 (15)	0.0347 (15)	0.0428 (17)	0.0008 (12)	0.0025 (13)	0.0038 (12)
C10	0.0540 (18)	0.0444 (17)	0.0403 (17)	0.0031 (14)	0.0057 (15)	0.0070 (14)
C11	0.061 (2)	0.0542 (19)	0.0453 (19)	0.0124 (16)	0.0133 (16)	0.0009 (15)
Cl1	0.0550 (5)	0.0464 (5)	0.0695 (6)	0.0110 (4)	0.0128 (4)	0.0129 (4)
N1	0.0591 (18)	0.0398 (16)	0.086 (2)	0.0005 (13)	0.0067 (17)	0.0113 (16)
O1	0.0863 (18)	0.0700 (17)	0.0639 (16)	0.0429 (14)	0.0108 (14)	0.0016 (13)
O2	0.0823 (18)	0.0523 (15)	0.0787 (18)	0.0170 (13)	0.0176 (14)	0.0166 (13)
O3	0.114 (2)	0.0672 (17)	0.0640 (18)	−0.0037 (16)	0.0037 (16)	0.0161 (14)
O4	0.225 (5)	0.077 (2)	0.064 (2)	0.008 (3)	0.010 (2)	−0.0115 (17)
O5	0.059 (2)	0.121 (3)	0.360 (7)	0.031 (2)	0.062 (3)	0.132 (4)

### Geometric parameters (Å, °)

**Table:**

C1—N1	1.318 (5)	C7—C9	1.429 (4)
C1—C2	1.359 (5)	C8—O1	1.369 (4)
C1—H1A	0.9300	C8—C11^i^	1.401 (5)
C2—C3	1.391 (4)	C9—C10	1.408 (4)
C2—H2A	0.9300	C9—C9^i^	1.424 (6)
C3—C4	1.383 (4)	C10—C11	1.364 (4)
C3—C6	1.504 (4)	C10—H10A	0.9300
C4—C5	1.363 (5)	C11—C8^i^	1.401 (5)
C4—H4A	0.9300	C11—H11A	0.9300
C5—N1	1.320 (5)	Cl1—O5	1.374 (3)
C5—H5A	0.9300	Cl1—O4	1.407 (4)
C6—C7	1.511 (4)	Cl1—O3	1.409 (3)
C6—H6A	0.9700	Cl1—O2	1.421 (3)
C6—H6B	0.9700	N1—H1B	0.8600
C7—C8	1.370 (4)	O1—H1C	0.8200
			
N1—C1—C2	120.5 (3)	C9—C7—C6	121.1 (3)
N1—C1—H1A	119.7	O1—C8—C7	117.7 (3)
C2—C1—H1A	119.7	O1—C8—C11^i^	121.1 (3)
C1—C2—C3	119.9 (3)	C7—C8—C11^i^	121.2 (3)
C1—C2—H2A	120.1	C10—C9—C9^i^	118.5 (3)
C3—C2—H2A	120.1	C10—C9—C7	122.0 (3)
C4—C3—C2	117.3 (3)	C9^i^—C9—C7	119.5 (3)
C4—C3—C6	121.5 (3)	C11—C10—C9	121.2 (3)
C2—C3—C6	121.2 (3)	C11—C10—H10A	119.4
C5—C4—C3	120.2 (3)	C9—C10—H10A	119.4
C5—C4—H4A	119.9	C10—C11—C8^i^	120.3 (3)
C3—C4—H4A	119.9	C10—C11—H11A	119.8
N1—C5—C4	120.1 (4)	C8^i^—C11—H11A	119.8
N1—C5—H5A	119.9	O5—Cl1—O4	111.5 (3)
C4—C5—H5A	119.9	O5—Cl1—O3	110.3 (3)
C3—C6—C7	113.1 (2)	O4—Cl1—O3	105.5 (2)
C3—C6—H6A	109.0	O5—Cl1—O2	109.5 (2)
C7—C6—H6A	109.0	O4—Cl1—O2	109.1 (2)
C3—C6—H6B	109.0	O3—Cl1—O2	110.97 (17)
C7—C6—H6B	109.0	C1—N1—C5	122.0 (3)
H6A—C6—H6B	107.8	C1—N1—H1B	119.0
C8—C7—C9	119.3 (3)	C5—N1—H1B	119.0
C8—C7—C6	119.6 (3)	C8—O1—H1C	109.5
			
N1—C1—C2—C3	0.3 (5)	C9—C7—C8—C11^i^	1.5 (5)
C1—C2—C3—C4	0.4 (4)	C6—C7—C8—C11^i^	−179.4 (3)
C1—C2—C3—C6	−179.2 (3)	C8—C7—C9—C10	178.7 (3)
C2—C3—C4—C5	−0.3 (5)	C6—C7—C9—C10	−0.4 (4)
C6—C3—C4—C5	179.2 (3)	C8—C7—C9—C9^i^	−0.1 (5)
C3—C4—C5—N1	−0.4 (5)	C6—C7—C9—C9^i^	−179.2 (3)
C4—C3—C6—C7	−135.8 (3)	C9^i^—C9—C10—C11	−1.1 (5)
C2—C3—C6—C7	43.7 (4)	C7—C9—C10—C11	−179.9 (3)
C3—C6—C7—C8	−96.3 (3)	C9—C10—C11—C8^i^	−0.3 (5)
C3—C6—C7—C9	82.8 (3)	C2—C1—N1—C5	−1.1 (5)
C9—C7—C8—O1	−178.5 (3)	C4—C5—N1—C1	1.1 (5)
C6—C7—C8—O1	0.6 (5)		

Symmetry codes: (i) −*x*, −*y*+1, −*z*.

### Hydrogen-bond geometry (Å, °)

**Table:**

*D*—H···*A*	*D*—H	H···*A*	*D*···*A*	*D*—H···*A*
O1—H1C···O2^ii^	0.82	2.05	2.859 (4)	169
N1—H1B···O3^iii^	0.86	2.24	2.997 (4)	148
N1—H1B···O4^iii^	0.86	2.33	3.121 (5)	153

Symmetry codes: (ii) −*x*+1, −*y*+1, −*z*; (iii) *x*−1, *y*−1, *z*.
